# Bacterial Community Composition in the Gut Content and Ambient Sediment of Sea Cucumber *Apostichopus japonicus* Revealed by 16S rRNA Gene Pyrosequencing

**DOI:** 10.1371/journal.pone.0100092

**Published:** 2014-06-26

**Authors:** Fei Gao, Fenghui Li, Jie Tan, Jingping Yan, Huiling Sun

**Affiliations:** Key Laboratory of Sustainable Development of Marine Fisheries, Yellow Sea Fisheries Research Institute, Chinese Academy of Fishery Sciences, Qingdao, PR China; J. Craig Venter Institute, United States of America

## Abstract

The composition of the bacterial communities in the contents of the foregut and hindgut of the sea cucumber *Apostichopus japonicus* and in the ambient surface sediment was surveyed by 16S rRNA gene 454-pyrosequencing. A total of 188,623 optimized reads and 15,527 operational taxonomic units (OTUs) were obtained from the ten gut contents samples and four surface sediment samples. The sequences in the sediments, foregut contents, and hindgut contents were assigned to 38.0±4.7, 31.2±6.2 and 27.8±6.5 phyla, respectively. The bacterial richness and Shannon diversity index were both higher in the ambient sediments than in the gut contents. *Proteobacteria* was the predominant phylum in both the gut contents and sediment samples. The predominant classes in the foregut, hindgut, and ambient sediment were *Holophagae* and *Gammaproteobacteria, Deltaproteobacteria* and *Gammaproteobacteria*, and *Gammaproteobacteria* and *Deltaproteobacteria*, respectively. The potential probiotics, including sequences related to *Bacillus*, lactic acid bacteria (*Lactobacillus*, *Lactococcus,* and *Streptococcus*) and *Pseudomonas* were detected in the gut of *A. japonicus*. Principle component analysis and heatmap figure showed that the foregut, hindgut, and ambient sediment respectively harbored different characteristic bacterial communities. Selective feeding of *A. japonicus* may be the primary source of the different bacterial communities between the foregut contents and ambient sediments.

## Introduction

The holothurians, or sea cucumbers, belong to the echinodermata, and is the general name of the class Holothuroidea. There are about 1200 species of sea cucumbers in the world [1], all of which are marine species. They obtain food by the ingestion of marine sediment, or by filtration of sea water [2]. Aspidochirote holothurians have shield-shaped retractile tentacles surrounding their mouths, and they are all deposit-feeding animals [3]. They ingest surface sediments containing microorganisms, meiofauna, decaying organic debris, inorganic components, and dissolved organic matter [4], [5], [6], [7], [8]. As the sediments are diluted by inorganic components and indigestible organic matter, they face the formidable task of gathering digestible resources [9].

Because of the high abundance, production and nutritional value, the bacteria in the sediment are considered either a direct food source or a source that indirectly provide the host with essential nutrients that are not otherwise available [5], [10], [11], [12], [13]. A study using fatty acid biomarkers has shown that bacteria are important dietary component of the sea cucumber *Apostichopus japonicus* [13]. The study of Moriarty [5] showed the sea cucumbers *Holothuvia atva* and *Stichopus chlovonotus* had a high assimilation efficiencies for bacteria averaging 30–40%. In *Holothuria tubulosa*, the assimilation efficiency for bacteria was found to be about 70% on an annual average, which was higher than particulate organic nitrogen and particulate carbohydrates [14]. Moreover, gut microbiota in holothurians *A. japonicus* and *Holothuria leucospilota* were shown to produce extracellular enzymes that could degrade indigestible breakdown products such as polysaccharides [15], [16], [17]. Other reports have suggested that gut bacteria may play a role in the supply of specific essential amino acids to the holothurians [18], [19].

The sea cucumber *A. japonicus* (Selenka) (*Echinodermata*, *Holothuroidea*, *Aspidochirotida*), is an epibenthic deposit-feeding species that mainly inhabits in northern-western Pacific, including the Bohai sea, the Yellow sea, east coast of Russia and coast of Japan and Korea [3]. Now, it is one of the most economically important holothurian species in coastal aquaculture and stock enhancement in China. Composition and diversity of microorganisms in the digestive tract of *A. japonicus* have been surveyed sporadically. Researchers have investigated the microorganism composition in the digestive tract of *A. japonicus* using traditional culture-dependent methods [15], [20], [21]. The bacterial diversity in the digestive tract of *A. japonicus* cultured in a pond was surveyed by Polymerase Chain Reaction-Denaturing Gradient Gel Electrophoresis (PCR-DGGE) based on 16S rDNA V3 fragments [8], and *Alphaproteobacteria*, *Gammaproteobacteria*, *Deltaproteobacteria*, *Bacteroidetes,* and *Mollicutes* were found to be the dominant bacteria. However, some studies have demonstrated that bacterial populations revealed using DGGE can represent less than 1% of the total community [22], [23]; therefore, the diversity of the bacterial communities in the digestive tract of *A. japonicus* may have been underestimated.

In contrast, massively parallel pyrosequencing technology can generate hundreds of thousands of short sequences from hypervariable regions in rRNA genes [24], [25], which increases the molecular sampling effort that is required to detect low abundance taxa. The powerful method that is noncultivation based has revolutionized microbial ecology. Furthermore, this technology makes it possible to analyze the total microbiota down to the genus level, even in large cohorts [26], [27], [28]. This allows for a more comprehensive exploration of the gut microbiota and its effects on the host. In recent years, pyrosequencing technology has been used to study the gut microbiota of many organisms including human, cow, termite, and grass carp [27], [29], [30]. However, to our knowledge, no reports of large molecular surveys of microbial communities in the gut of holothurians have been published.

Holothurians are a prominent group of deposit-feeders. They rework considerable amounts of sediments, and their activity may influence microbial processes at the sediment-water interface. In this study, we surveyed the bacterial community composition in the habitat surface sediment, the foregut and hindgut contents of *A. japonicus* by barcoded 16S rRNA gene 454-pyrosequencing. Comparisons among the bacterial community structures in sediments ingested by holothurians and in the surrounding sediments may provide evidence about the feeding strategy of the animals, which may help reveal the association between their gut microbiota and environmental habitat. A comprehensive investigation of the normal gut microbiota associated with the sea cucumber will help shed light on the bacteria that play important roles in the physiology and nutrition of their host.

## Materials and Methods

### Sample collection

In the bottom enhancement area, sea cucumbers feed only on natural diets and are not treated with antibiotics. Thus, the bacterial composition of the sea cucumbers there are likely to be more similar to the natural population. In this study, we sampled sea cucumbers from a bottom enhancement area (120°41′N, 36°15′E) in Qingdao, Shandong Province, China. Five sea cucumbers with an average weight of 127.73±3.49 g were collected, on December 3, 2011 when the water temperature was 12°C. At that time, the sea cucumbers had completely recovered from the long aestivation phase, and fed normally in typical active phase [31]. Surface sediments (0–2 cm) were collected separately from four locations around the sampled sea cucumbers (the distance<20 cm) using syringe samplers (capacity 50 ml) [32]. The sampled animals were immediately stored on ice and transported to the laboratory within one hour of collection.

Before dissection, the surface skin of the sea cucumbers was sterilized with 70% ethanol to reduce contamination. The ventral surface was dissected with a sterile scalpel to expose the body cavity. The foregut and hindgut contents were squeezed out separately and gathered in sterile freezing tubes. In order to decrease the reproduction of specific microbes in guts, only the contents in the anterior part of the foregut, about 2–3 cm, were taken as the foregut contents samples. The contents in each gut section were squeezed out carefully making sure that the gut tissue and bacteria adhering to it were excluded. The careful sampling avoided the inclusion of the autochthonous bacteria in the contents samples. The gut contents and ambient sediments samples were stored at –80°C until required for analysis.

### Ethics statement

Not applicable. Our research did not involve human participants or samples. No specific permits were required for the described field studies.

### DNA extraction and PCR amplification

DNA was extracted from the gut contents and sediment samples using the E.Z.N.A Soil DNA Kit (Omega Bio-Tek, Norcross, GA, USA) according to the manufacturer’s protocol.

The universal primer set, 8F: 5′-AGAGTTTGATCCTGGCTCAG-3′ and 533R: 5′-TTACCGCGGCTGCTGGCAC-3′, was used for amplification of the V1–V3 region of 16S rDNA from the gut contents and sediments [33], [34]. Barcodes that allow sample multiplexing during pyrosequencing were incorporated between the 454 adapter and the forward primer. The 20 µL reaction mixture contained 1 U of FastPfu DNA polymerase, 4 µL of 5×FastPfu reaction buffer, 2 µL of 2.5 mM dNTPs mixture, 0.4 µL of each primer (5 µM), and 10 ng of genomic DNA. The thermocycling steps were as follows: 95°C for 2 min, followed by 25 cycles at of 95°C for 30 sec, 55°C for 30 sec, 72°C for 30 sec, and a final extension step at 72°C for 5 min. PCR amplicons were purified using a DNA gel extraction kit (Axygen, China).

### High-throughput 454-pyrosequencing

The DNA concentration of each PCR product was determined using a Quant-iT PicoGreen double-stranded DNA assay (Invitrogen, Germany). Following quantitation, the amplicons were subjected to emulsion PCR to generate amplicon libraries, as recommended by 454 Life Sciences. Amplicons of the samples from gut contents and surrounding surface sediments were subjected to pyrosequencing on the Roche 454 GS FLX Titanium platform at Majorbio Bio-Pharm Technology Co., Ltd., Shanghai, China.

### Statistical and bioinformatics analysis

Data were analyzed using the SPSS 17.0 statistical software packages. All values are presented as the means ± standard deviation (mean±SD). The level of statistical significance was determined using a t-test. The statistical significance was set at p<0.05.

Barcodes and primers were checked for completeness, and the reads with complete barcode sequences were selected as valid sequences. Pyrosequencing reads with ambiguous bases, more than six repeated single bases, more than two mismatched bases in the primers, and reads shorter than 150 bp were removed. The average length of the optimized reads was 476.6 bp.

Taxonomic classification of the reads was performed by matching them against sequences in the SILVA database (version 106; [34], [35]). Mothur software was used to classify the sequences (http://www.mothur.org/wiki/Main_Page). The matrices were used to define the number of operational taxonomic units (OTUs) at sequence divergences of 3% in the communities [34]. The 16S rRNA gene sequences derived from pyrosequencing have been deposited in the NCBI Sequence Read Archive under accession number SRP038895.

The coverage percentage was estimated by Good’s method [36]. The abundance-based coverage estimator (ACE), bias-corrected Chao1 richness estimator, and the Shannon and Simpson diversity indices were also calculated [34]. Good’s coverage percentage was calculated as [1–(*n*/*N*)]×100, where *n* represents the number of single-member phylotypes and *N* represents the number of sequences. All the analyses were performed using the mothur program (http://www.mothur.org; [34,37,38). Rarefaction analysis was also performed using the mothur program. Heatmaps were generated with the R-package gplots. The microbial community structures in different samples were compared using UniFrac based on the phylogenetic relationship of representative reads from different samples [39]. UniFrac PCA was used for the principal component analysis (PCA) [37], [38], [39].

## Results

A total of 188,623 optimized reads were obtained from the ten gut contents samples (the foregut and hindgut contents samples from the five sea cucumber) and four surface sediment samples by the 454-pyrosequencing. The optimized read numbers for each sample ranged from 9,606 to 16,030 with a mean average of 13,473±2,181.

### Richness and Diversity

A total of 15,527 OTUs were obtained from the 14 samples. The foregut contents samples (GCA1–GCA5) from each of the five sea cucumbers contained from 1,725 to 2,623 OTUs, and the hindgut contents samples (GCP1–GCP5) from each of the five sea cucumbers contained 1,975 to 2,342 OTUs. The four surface sediments (SED1–SED4) contained 2,533 to 4,524 OTUs (Table 1).

**Table 1:** Species richness estimates obtained at genetic distances of 3%.

Sample ID	OTU	Ace	Chao	Coverage (%)
GCA1	2623	5159	4313	92.3
		(4957,5379)	(4069,4598)	
GCA2	2518	6233	4556	90.3
		(5966,6521)	(4271,4887)	
GCA3	2600	5760	4617	88.7
		(5522,6017)	(4334,4947)	
GCA4	2499	4933	4231	92.8
		(4736,5147)	(3972,4534)	
GCA5	1725	3070	2634	95.0
		(2932,3224)	(2471,2833)	
GCP1	2289	4469	3803	93.3
		(4284,4672)	(3567,4082)	
GCP2	2128	3305	3184	91.7
		(3153,3479)	3017,3383)	
GCP3	2342	4721	4154	92.8
		(4526,4935)	(3872,4488)	
GCP4	1975	3921	3191	93.3
		(3746,4113)	(2989,3433)	
GCP5	2216	4054	3507	94.2
		(3889,4236)	(3299,3755)	
SED1	2685	6704	5215	86.2
		(6426,7004)	(4870,5614)	
SED2	2533	7463	4918	84.8
		(7131,7818)	(4595,5291)	
SED3	4324	9589	7611	85.2
		(9282,9914)	(7251,8014)	
SED4	4513	14905	9789	75.1
		(14370,15469)	(9258,10379)	

GCA, GCP respectively represent the content of foregut and hindgut in the five sapmpled sea cucumbers. For example, GCA1 represents the foregut content in the sea cucumber No. 1. SED stands for the four surface sediments samples. The numbers in the brackets of Ace, Chao and Coverage lines were the upper limit value and lower limit value.

**Figure 1:**
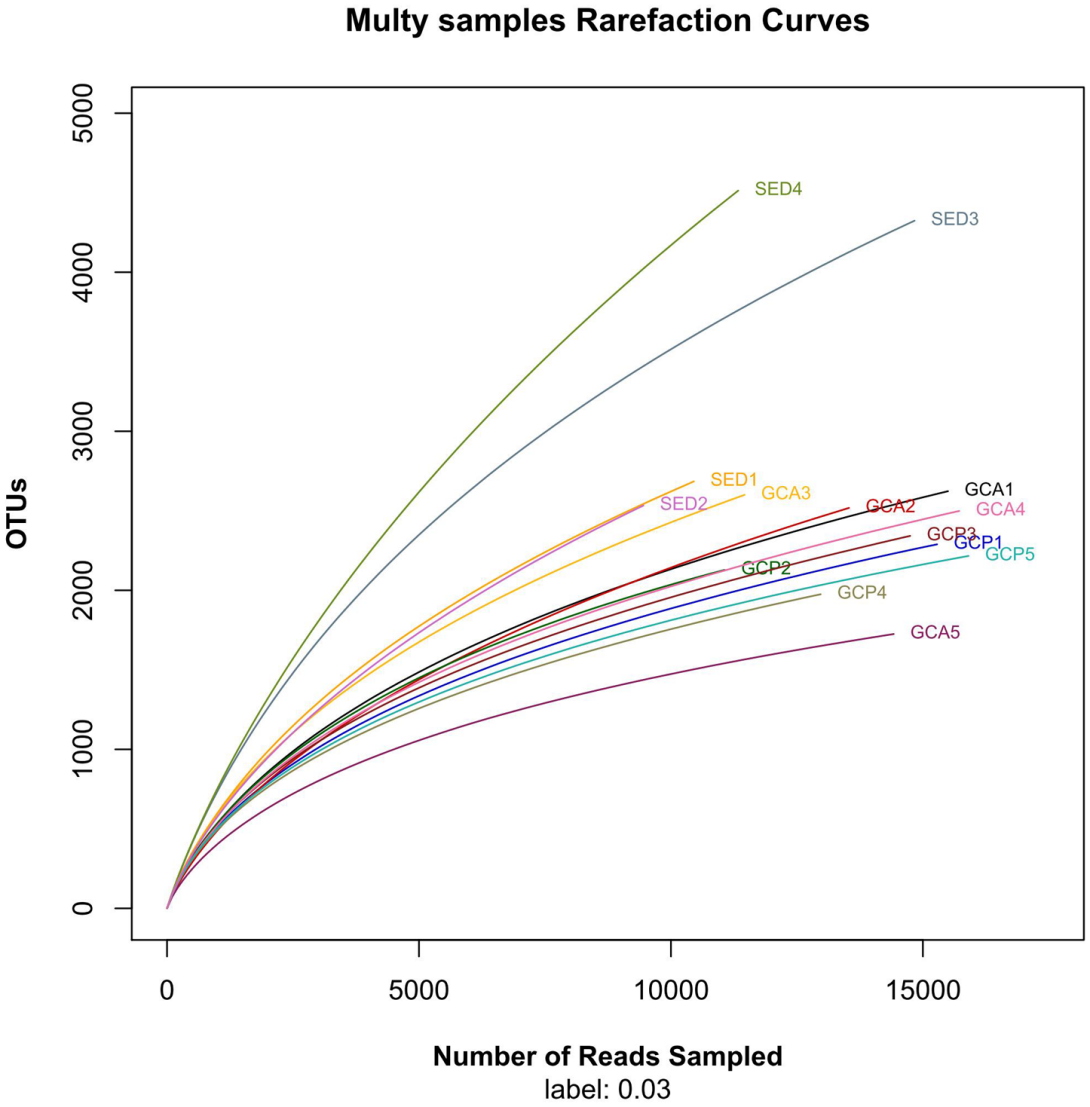
Rarefaction analysis of the gut content and sediment samples. Rarefaction curves of OTUs clustered for a dissimilarity of 3%. Abbreviations are as in table 1.

The Good’s coverage of all the gut content samples was estimated to be between 88.7% and 95.0%; the coverage in all the sediment samples was over 84.8% (except for SED4 with 75.1% coverage, Table 1). The rarefaction curves based on the OTUs (Fig. 1) show that all the gut content samples and three of the sediments samples (SED1, SED2, and SED3) tended to approach the saturation plateau, while SED4 did not reach the plateau.

Richness diversity (Table 1) was calculated by estimating the number of OTUs based on the ACE and Chao1 values at the 3% dissimilarity levels. Among the foregut content samples (GCA1–GCA5), the ACE and Chao1 values in four of the samples (the exception was GCA1) were higher than the values in the hindgut content samples (GCP1–GCP5). The ACE and Chao1 values in all the sediment samples (SED1–SED4) were higher than the values in all the foregut and hindgut content samples (Table 1). The ACE and Chao1 values indicated that the bacterial richness in the SED samples was higher than the richness in the GCA and GCP samples, and that the richness of bacteria in GCA samples was higher than it was in the GCP samples.

**Figure 2:**
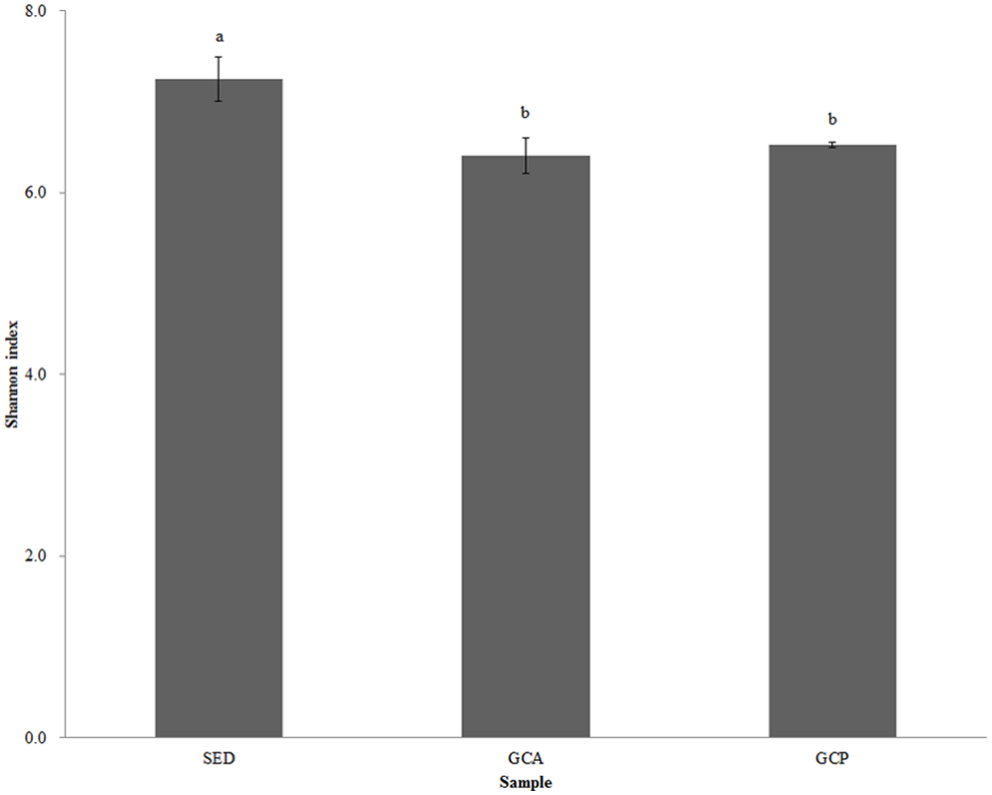
Shannon index of the different samples. Abbreviations are as in table 1.

The Shannon diversity indices in the SED samples were significantly higher than the indices in the GCA and GCP samples (p<0.05, Fig. 2). The results suggested that the bacterial diversity in the habitat sediments was higher than the diversity in the gut contents of the sea cucumbers.

### Bacterial community structure in the gut contents and the sediments

An average of 38.0±4.7, 31.2±6.2 and 27.8±6.5 different phyla were identified from the ambient sediment, foregut content, and hindgut content of the sea cucumbers, respectively. The reads from the SED libraries contained a higher proportion of unclassified bacteria (24.33–27.69%) than the reads from the gut content libraries (21.04–27.11% from GCA and 14.14–18.03% from GCP).

**Figure 3:**
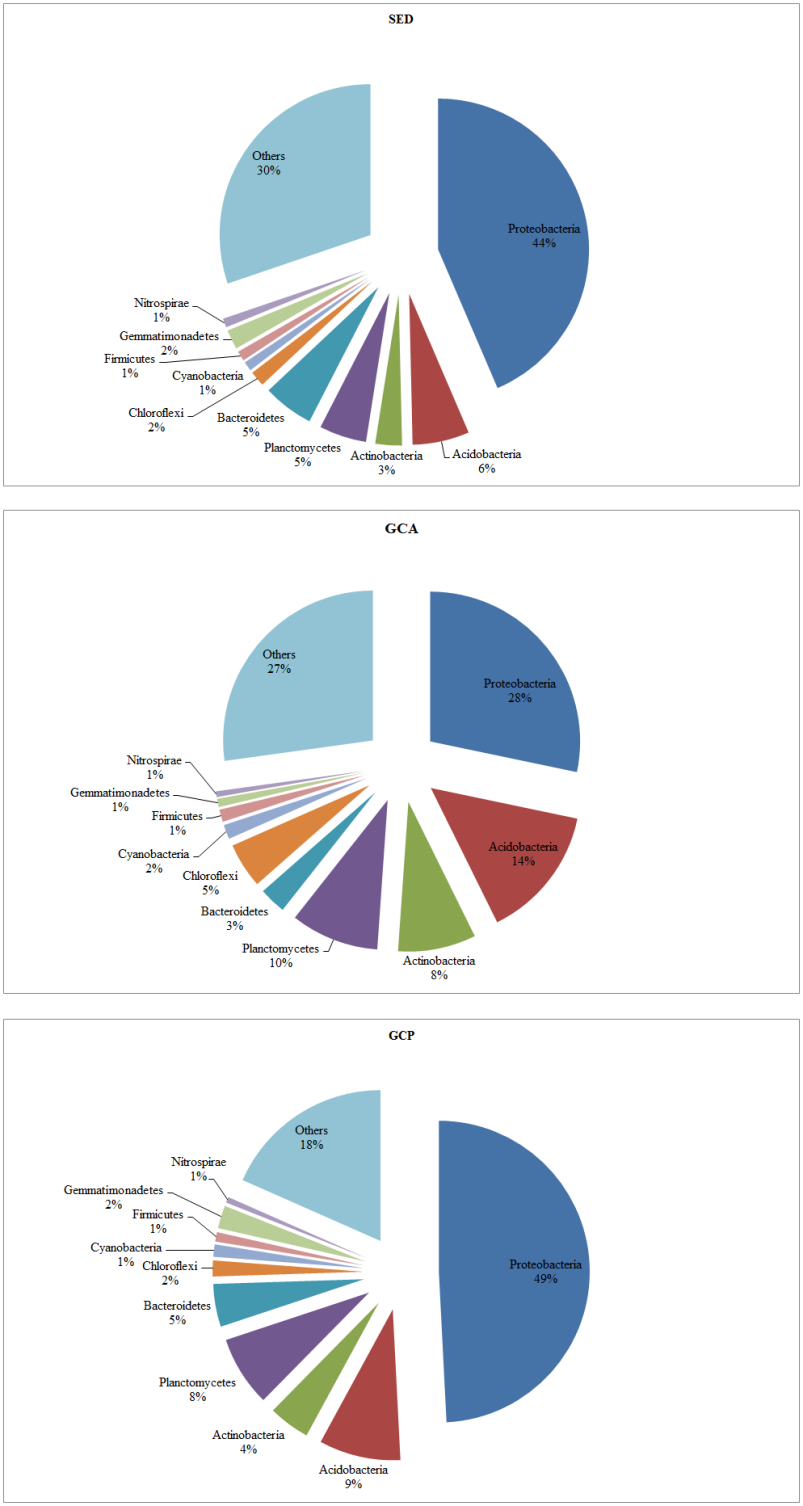
Relative read abundance of different bacterial phyla above a cutoff value of 0.6%. Abbreviations are as in table 1.

Based on an abundance cutoff of 0.6%, the prevalent phyla accounted for 69.79%, 72.82%, and 81.62% of the reads in the SED, GCA, and GCP libraries respectively (Fig. 3). *Proteobacteria* was the predominant phylum, making up 43.55±1.76%, 28.28±5.60%, and 49.19±1.68% of the reads in the SED, GCA, and GCP libraries respectively. The abundance of *Proteobacteria* in the foregut contents was significantly lower than the abundances in the surrounding sediments and in the hindgut contents (p<0.05). Conversely, the abundances of *Acidobacteria*, *Actinobacteria*, *Planctomycetes*, and *Chloroflexi* in the foregut contents were significantly higher than they were in the sediments (p<0.05), while the abundances of *Acidobacteria*, *Actinobacteria*, and *Chloroflexi* in the hindgut contents were significantly lower than their abundances in the foregut (p<0.05).

**Figure 4:**
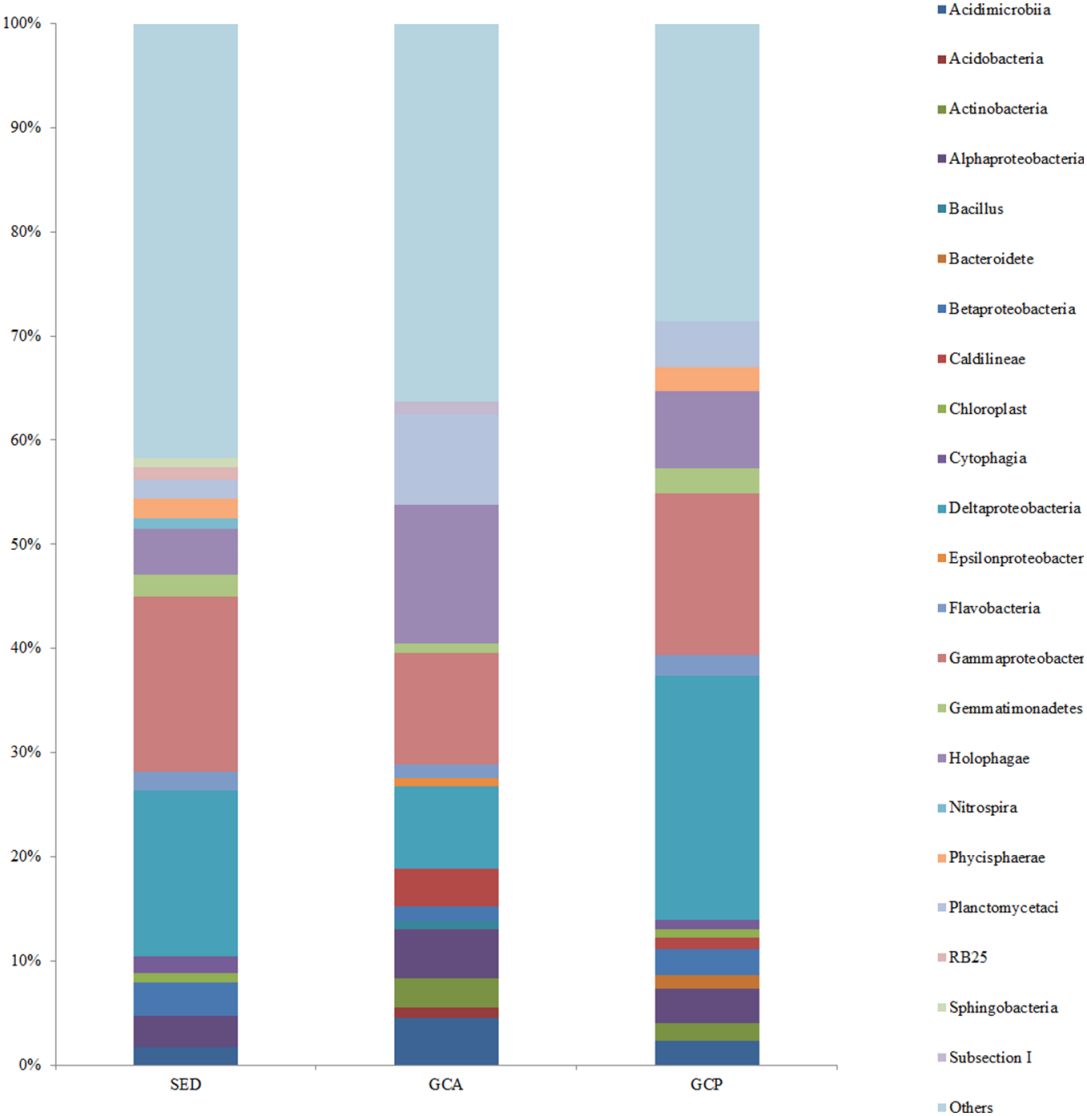
The relative abundance of the 15 most abundant classes. Abbreviations are as in table 1.

The reads from the SED, GCA, and GCP libraries fell into 71.3±14.2, 60.8±13.0, and 51.6±1.4 different classes, respectively. The 15 most abundant classes in the different samples accounted for 52.77%, 59.17%, and 65.62% of the reads in the SED, GCA, and GCP libraries respectively (Fig. 4). In the SED libraries, the predominant classes were *Gammaproteobacteria* (16.81±0.73%) and *Deltaproteobacteria* (15.93±3.21%). The predominant classes in the GCA and GCP libraries were *Holophagae* (13.31±5.53%) and *Deltaproteobacteria* (23.44±2.66%), respectively. *Gammaproteobacteria* was the second most abundant class in both the GCA and GCP libraries (10.78±3.87% and 15.55±1.65%, respectively).

A large proportion (47.2–70.8%) of the reads in all the libraries could not be classified at the genus level. The most abundant genera, based on an abundance cutoff of 1%, in the GCA libraries were sequences related to unclassified *Caldilineaceae* (3.07±1.83%), unclassified *Desulfobulbaceae* (2.14±2.10%), JTB255_marine_benthic_group (uncultured *Gammaproteobacterium*, *Xanthomonadales*, *Sinobacteraceae*, 1.23±2.16%), Pir4_lineage (*Planctomycetes*, *Planctomycetacia*, *Planctomycetales*, *Planctomycetaceae*, 1.63±1.01%), *Planctomyces* (1.55±0.46%), *Rhodopirellula* (1.95±0.74%), and *Synechococcus* (1.15±0.54%). In the GCP libraries, the most abundant genera (relative abundance>1%) were *Desulfosarcina* (5.82±0.57%), JTB255_marine_benthic_group (2.99±1.06%), *Rhodopirellula* (1.34±0.43%), *Prolixibacter* (1.34±2.73%), and *Marinicella* (1.16±0.33%). In the SED libraries, *Cupriavid* (1.85±1.61%), *Desulfobulbus* (2.50±0.94%), JTB255_marine_benthic_group (4.74±0.60%), *Marinicella* (1.24±0.62%), and *Desulfosarcina* (2.47±0.35%) were the dominant genera (relative abundance>1%).

Gram-positive bacteria (*Bacillus*, *Carnobacterium*, *Enterococcus*, *Lactobacillus*, *Lactococcus*, *Micrococcus*, *Streptococcus*, and *Weissella*), and Gram-negative bacteria (*Aeromonas*, *Alteromonas*, *Photorhodobacterium*, *Pseudomonas*, and *Vibrio*) have been used successfully as probiotics in aquaculture [40]. In the present study, reads related to the genera *Bacillus*, *Lactobacillus*, *Lactococcus*, *Streptococcus*, *Pseudomonas*, and *Vibrio* were detected in the gut contents and sediments libraries, although these reads were in low abundance (Table 2). The genera *Micrococcus*, *Aeromonas*, and *Alteromonas* were detected only in the SED libraries (Table 2). *Enterococcus*, *Weissella*, and *Photorhodobacterium* were not found in any of the gut contents or sediments libraries (Table 2).

**Table 2:** Relative abundance (%) of candidate probiotics in the gut content and sediment samples.

Genus	GCA(%)	GCP(%)	SED(%)
*Bacillus*	0.043±0.021	0.015±0.007	0.039±0.016
*Carnobacterium*	NF	0.004±0.004	0.003±0.003
*Enterococcus*	NF	NF	NF
*Lactobacillus*	0.021±0.006	0.022±0.022	0.008±0.008
*Lactococcus*	0.043±0.021	0.110±0.085	0.073±0.031
*Micrococcus*	NF	NF	0.009±0.004
*Streptococcus*	0.152±0.046	0.236±0.188	0.193±0.062
*Weissella*	NF	NF	NF
*Aeromonas*	NF	NF	0.004±0.002
*Alteromonas*	NF	NF	0.002±0.002
*Photorhodobacterium*	NF	NF	NF
*Pseudomonas*	0.085±0.027	0.142±0.125	0.080±0.030
*Vibrio*	0.003±0.001	0.023±0.011	0.064±0.043

NF means not found any reads in the library. Abbreviations are as in table 1.

### Relationships among the bacterial communities from the gut content and the sediment samples

**Figure 5:**
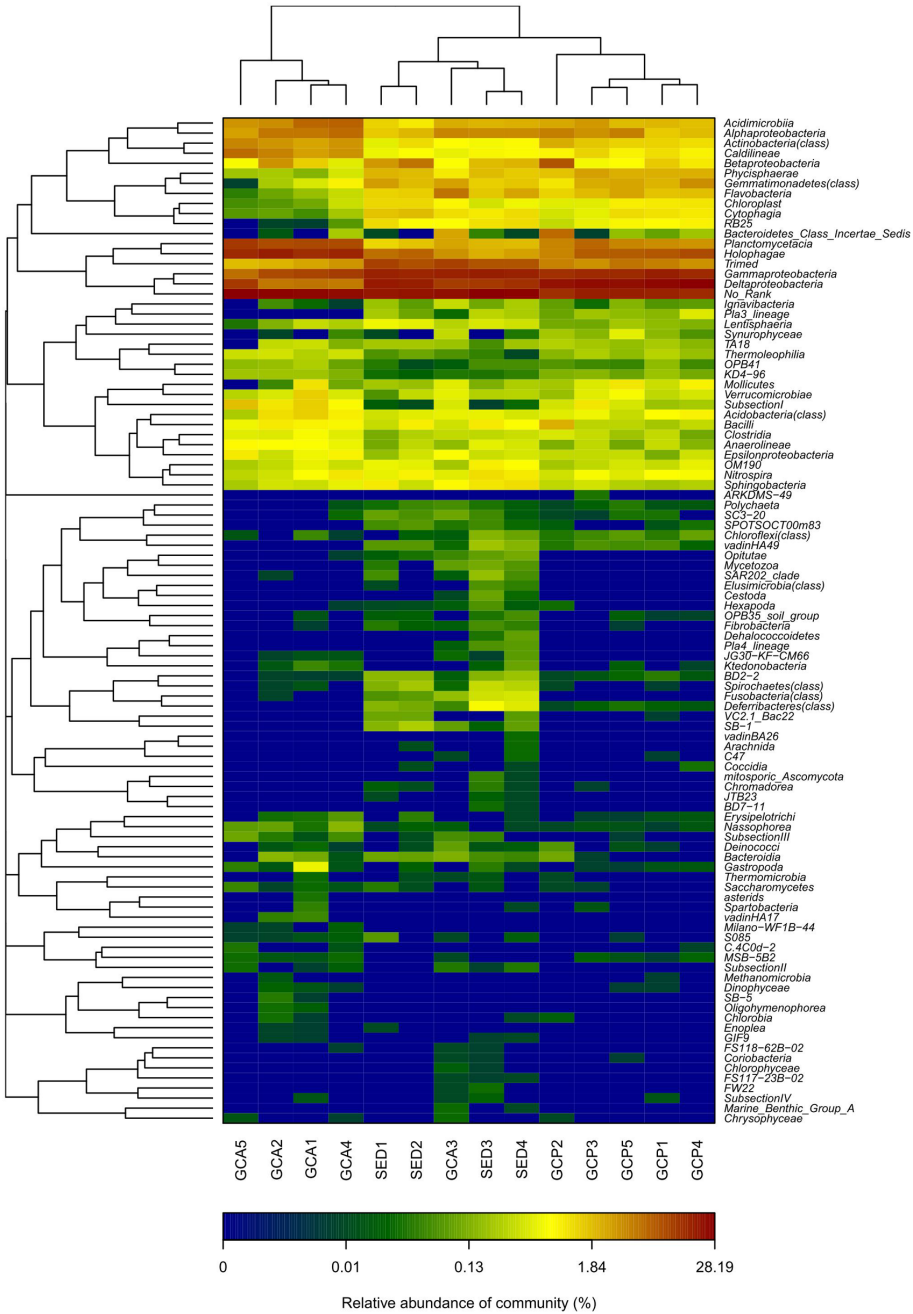
Hierarchically clustered heatmap of the bacterial distribution of different communities. Double hierarchical dendrogram shows the bacterial distribution. The bacterial phylogenetic tree was calculated using the neighbor-joining method and the relationship among samples was determined by Bray-Curtis distance and the complete clustering method. The heatmap plot depicts the relative percentage of each bacterial class (variables clustering on the vertical-axis) within each sample (horizon-axis clustering). The relative values for bacterial class are indicated by color intensity with the legend indicated at the bottom of the figure.

**Figure 6:**
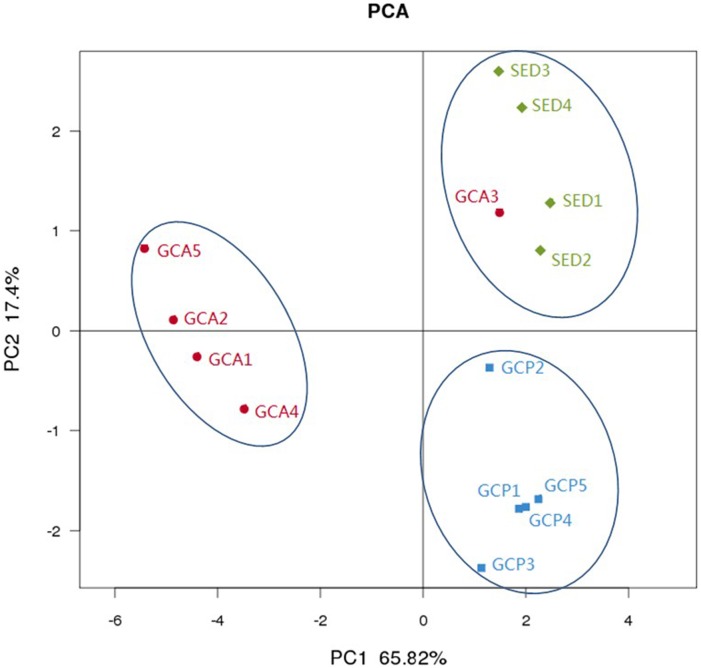
PCA results showing the relatedness of bacterial community in the different samples. The PCA plots were constructed with the weighted UniFracPCA method. Abbreviations are as in table 1.

A hierarchical clustering heat map analysis was performed at the class level based on the top 100 most abundant bacterial communities across all 14 samples. The analysis showed that, in general, the samples segregated into two groups (Fig. 5): one group was composed mainly of the foregut contents samples GCA1, GCA2, GCA4, and GCA5; and the other group was composed mainly of the hindgut contents samples (GCP1–GCP5) and the SED samples. In the second group, the SED samples and GCA3 formed one cluster, and all the GCP samples formed a second cluster. PCA was performed to reveal the relationships among the different samples. Three groups were distinguished, namely, all the GCP samples, all the GCA samples except for GCA3, and all the SED samples plus GCA3 (Fig. 6). The GCA samples were grouped on the left-hand side of the graph along the first principal component axis (PC1), which accounted for 65.82% of the total variations. The SED samples and the GCP samples were separated distinctly by the second principal component (PC2) rather than by PC1. PC2 accounted for 17.4% of the variance in the bacterial communities. Overall, the PC1 and PC2 axes explained 83.22% of the variations between the different bacterial communities. The results of the PCA and heat map analyses are in agreement. Both the analyses indicated that the foregut contents, hindgut contents, and sediments samples have different characteristic bacterial communities.

## Discussion

Previous studies have shown that 454-pyrosequencing has a higher capacity than PCR-DGGE and culture-dependent methods to explore bacterial richness, indicating that pyrosequencing is an efficient way to access microbial diversity [41]. This study is the first report in which a second-generation sequencing technology has been used to investigate the gut microbial community in a holothurian species with important economic value.

In the present study, each gut content library contained 1725 to 2623 OTUs, and 21 to 37 different phyla, which are much higher numbers than the numbers detected in any of the previous investigations of gut microbiota from *A. japonicus*. In the previous studies, the cultivable gut microorganisms of *A. japonicus* were reported bo belong to only two phyla (*Proteobacteria* and *Firmicutes*) [15], [21]; and in two PCR-DGGE studies only three and five phyla were found in the gut contents of *A. japonicus* cultured in a pond and a bottom enhancement area, respectively [8], [42]. Therefore, the results disclosed a complicated gut microbiota in the holothurian using 454-pyrosequencing method.

### Dominant bacteria in the gut contents

*Proteobacteria* and *Firmicutes* are the most dominant phyla detected in the intestinal contents of many fish species [38], [43], [44]. The present study suggests that *Proteobacteria* is the predominant phylum in the gut contents of the *A. japonicus*, and *Proteobacteria* was also proved to be the predominant phylum in the gut contents of *A. japonicus* cultured in a pond by PCR-DGGE method [8]. The dominant culturable bacteria in the gut of *A. japonicus* were also reported to be *Proteobacteria* [15], [21].

At the class level, *Gammaproteobacteria* was the predominant bacterial group in the gut contents of *A. japonicus* cultured in a pond and in a bottom enhancement area using PCR-DGGE [8], [42]. Furthermore, *Gammaproteobacteria* was also reported to be dominant in the digestive tract of *A. japonicus* by traditional culture-dependent methods [15]. Nevertheless, in the present study, the 454-pyrosequencing results showed that the predominant classes in the GCA and GCP libraries of the sea cucumber were *Holophagae* and *Deltaproteobacteria*, respectively, although *Gammaproteobacteria* was the second most abundant class in all the gut contents samples.

All the prevalent bacterial genera in gut contents of *A. japonicus* disclosed by the current study were never isolated from the same species of sea cucumber in previous studies [15], [21]. However, most of the isolated bacteria (*Pseudomonas*, *Vibrio*, *Bacillus*, *Shewanella*, *Aerococcus*, *Acinetobacter*, *Photobacterium*, *Staphylococcus*, *Pseudoalteromonas*) by culture-dependent method from the digestive tract of *A. japonicus* [15], [21] were detected in the present study. Vaz-Moreira et al. [41] proved that the culture-dependent diversity survey presented lower coverage than culture-independent methods (DGGE and 454-pyrosequencing). Furthermore, although the predominant bacterial phyla detected by the culture-dependent and culture-independent methods were the same, the sequence similarity analysis showed that different OTUs were targeted by the two methods [41]. A similar finding was reported in a survey of carp gut microbiota [45]. The incongruence in different taxonomy levels may be caused by biases introduced by the different methods. So far, most microorganisms in the natural environment cannot be easily isolated and cultivated on traditional agar substrates [46], [47], which may lead to biased results. Another possible bias is the preferential amplification of some DNA fragments by high throughput sequencing method [41].

The sulfate-reducing bacteria (SRB) *Desulfosarcina* were the predominant bacteria in the hindgut contents of *A. japonicus*. *Desulfosarcina* has also been found in the intestinal tracts of humans and other terrestrial animals [48], [49], [50]. SRB in the intestinal tract may participate in acetate production and nitrogen fixation [50], [51], [52], and thus provide nutrition for their hosts. SRB has been found mainly in marine sediments, freshwater sediments, and in sewage sludge [53], [54], [55], and are of great importance in the final step of carbon recycling and in the sulfur cycle in anaerobic environments [50]. In the present study, the abundance of SRB, including *Desulfobulbus* and *Desulfosarcina*, was high in the surrounding surface sediment of the sea cucumber. In anaerobic marine sediments, SRB can catalyze more than 50% of the total carbon oxidation [53].

### Candidate probiotics

Probiotics have been used widely in aquaculture as biological control agents against animal disease or as activators of nutrient intake by the host [56], [57]. Successful colonization in the digestive tract is often considered a prerequisite for dietary probiotics, and the candidate strains should come preferably from the host gut microbiota [45]. However, no research to investigate the application of probiotics to sea cucumber culture has been reported until now. The genus *Bacillus* contains Gram-positive rods that form a single endospore and represent a peculiar case among the bacteria used as probiotics [58]. In the current study, *Bacillus* sp. was present in all the gut contents samples. Lactic acid bacteria (*Lactobacillus*, *Lactococcus,* and *Streptococcus*) and *Pseudomonas* are also important biological control agents in aquaculture [59]. In this study, these bacteria were detected in the gut of *A. japonicus*, suggesting that they could be used as candidate probiotics.

### Relationship between bacterial communities in the foregut contents and the sediments

In the current study, we compared the bacterial community composition in the foregut contents of *A. japonicus* and the surrounding sediments. Our dataset indicated higher bacteria richness is present in the habitat sediments than foregut contents of the sea cucumbers. The Shannon diversity indices for the sediments were also found to be significantly higher than the indices in the foregut contents. The abundances of the dominant bacteria *Proteobacteria*, *Acidobacteria*, *Actinobacteria*, *Planctomycetes*, and *Chloroflexi* in the foregut contents were significantly different from their abundances in the surrounding sediments. Furthermore, the PCA score plot and heatmap figure showed that the foregut content samples of *A. japonicus* and the surrounding sediment samples clustered in different groups. Together, these results showed that the foregut content and the surrounding sediment contained different characteristic bacterial communities.

The different bacterial communities in the sediments and foregut contents of *A. japonicus* could have three possible sources: 1) selective feeding by the sea cucumber; 2) reproduction of specific microbes in the gut; and 3) microflora attached to the gut wall. In this study, we tried to decrease the effects of the latter two factors while extracting the samples from the gut. Only the contents in the anterior part of foregut (about 2–3 cm) were sampled as the foregut contents so as to decrease the effect of specific microbes reproduction in guts. Because the foregut is a simple tube through which food material passes, there would not be much time for bacteria to grow there, and, if organic matter which supported rapid bacterial growth was present, it is more likely that bacteria in the sediment would use it before it became available to bacteria in the gut [5]. Therefore, it is most unlikely that the difference between foregut microbes and surrounding microbes was due to the reproduction of specific microbes in foregut. To reduce the possible autochthonous bacteria sources, the gut contents were squeezed out carefully making certain that gut tissue and contents adhering to it were excluded while sampling. Therefore, the different bacterial communities in the foregut contents and sediments may be caused mainly by the selective feeding of *A. japonicus*.

To describe the selectivity in sediment feeders, it is usual to compare certain parameters of the foregut content with the corresponding parameters in the ambient sediment in previous study. For example, Moriarty [5] found a significantly higher bacterial biomass in the foregut contents of holothurians compared with the bacterial biomass in the sediment, and commented that the *Holothuvia atva* and *Stichopus chlovonotus* selected sediment fractions that had a high bacterial content. Witbaard et al. [60] showed that concentrations of chlorophyll a in the foregut contents of holothurian *Oneirophanta mutabilis* collected from the Porcupine Abyssal Plain in the north-east Atlantic Ocean were 5 to 15 times higher than in surface sediments, which implying that it fed selectively on fresh material.

In spite of the present finding, a previous study using PCR-DGGE disclosed that the bacterial community structures in the foregut contents and the surrounding sediments of *A. japonicus* share a low similarity [42]. In addition, Sun and Chen [15] found that heterotrophic bacteria in the foregut contents were about 30 times more abundant than in the surrounding sediments. In this study, *Bacillus* was found to be abundant in the surrounding sediment, but was barely detectable in the gut contents of *A. japonicus* [15]. Moreover, the abundance of bacteria capable of decomposing sodium alginate and chitin was 12.23 and 7.45 times higher in gut contents than in the surrounding sediments, respectively. Based on the current and previous studies, we propose that *A. japonicus* may ingest its diet selectively from the benthic environment. Selective feeding feature has also been reported for some species of holothurians in previous study [61], [62], [63], [64], [65]. However, without a well-controlled aquarium environment, other factors, such as the digestion of the host, may also play a role in the un-resemblances of the bacterial communities between the foreguts and the surrouding sediments. A well-controlled experiment shoud be done to prove the selective-feeding feature of the animal in the future study.

## Conclusion

Here, the complicated gut microbial community information for the holothurian *A. japonicus* was obtained using the high-throughput 16S-based molecular microbiology method. The potential probiotics, including sequences related to *Bacillus*, lactic acid bacteria (*Lactobacillus*, *Lactococcus,* and *Streptococcus*) and *Pseudomonas* were detected in the gut of *A. japonicus*. The foregut contents, hindgut and ambient sediments harbored different characteristic bacterial communities. Selective feeding of *A. japonicus* were proposed to be the primary source of the different bacterial communities between the foregut contents and ambient sediments.
